# Hydroxyzine-Induced Torsade De Pointes: A Case Report and a Literature Review

**DOI:** 10.7759/cureus.41588

**Published:** 2023-07-09

**Authors:** Muhammad Adil Afzal, Noman Khalid, Muhammad Abdullah, Ata ul-Haiy, Patrick Michael

**Affiliations:** 1 Internal Medicine, St. Joseph's University Medical Center, Paterson, USA; 2 Public Health and Community Medicine, Shaikh Khalifa Bin Zayed Al-Nahyan Medical and Dental College, Shaikh Zayed Federal Postgraduate Medical Institute at Shaikh Zayed Medical Complex, Lahore, PAK; 3 Internal Medicine, Mayo Hospital, Kind Edward Medical University, Lahore, PAK

**Keywords:** opioid withdrawal, polymorphic ventricular tachycardia, qtc prolongation, torsades de pointes, hydroxyzine

## Abstract

Hydroxyzine is an H1-receptor antagonist used for managing allergies, anxiety, opioid withdrawal, and insomnia. An adverse effect of hydroxyzine, QT prolongation, may lead to torsade de pointes (TdP). Our case report and literature review highlight the risk of TdP with hydroxyzine use. Our patient, a 58-year-old male with an implantable cardioverter defibrillator (ICD) and a history of polysubstance abuse presented with chest pain and shortness of breath. During the admission, the patient started experiencing symptoms of opioid withdrawal, which were refractory to buprenorphine. Hydroxyzine 50 mg was administered as recommended for symptomatic anxiety relief. Overnight the patient developed TdP, which was managed by MgSO_4_, amiodarone, and lidocaine, but did not resolve the arrhythmia. The patient was sedated and intubated, which led to the episode's resolution. This case report and literature review underscore the importance of cautious prescribing practices for hydroxyzine and other QT-prolonging drugs to prevent TdP. Healthcare providers should conduct personalized risk assessments, monitor electrolyte levels, and perform regular electrocardiograms. Administering the lowest effective dose, avoiding drug interactions, and exercising caution in patients with underlying repolarization abnormalities or a history of TdP are crucial. These measures help minimize the risk of TdP associated with low-dose hydroxyzine therapy.

## Introduction

Hydroxyzine, a potent H1-receptor antagonist, is widely prescribed for managing allergic reactions, anxiety, and insomnia, and as a sedative during medical procedures [1]. Although the exact mechanism of its sedative effect remains unclear, it is believed to involve central anticholinergic activity [2]. In addition, hydroxyzine has been commonly used as adjunctive therapy with buprenorphine/naltrexone to manage opioid withdrawal symptoms [3].

The association between hydroxyzine and the risk of ventricular tachyarrhythmias, specifically due to QT interval prolongation, has been reported in the literature [4,5]. Among antihistamines, hydroxyzine is known to have a dose-dependent effect on cardiac repolarization, leading to QT interval prolongation [1]. Although rare, hydroxyzine-induced ventricular tachycardias (VTs), such as polymorphic VT (PVT) and torsade de pointes (TdP), have been documented and can result in life-threatening arrhythmias and sudden cardiac arrest [6].

This report presents a rare case of hydroxyzine-induced QT prolongation that rapidly deteriorated into TdP. Through this case and a comprehensive review of the existing literature, we aim to underscore the importance of recognizing and anticipating these potentially life-threatening complications associated with hydroxyzine use. By raising awareness of these risks, we aim to promote informed prescribing practices and mitigate the occurrence of undesirable outcomes.

## Case presentation

A 58-year-old male with a complex history presented to the emergency department (ED) complaining of shortness of breath and chest pain. The patient had a past medical history of polysubstance abuse, including heroin, hypertension, heart failure with preserved ejection fraction, prolonged QTc, and TdP (status post automatic implantable cardioverter defibrillator (AICD)). It was reported that the patient was found in a room full of smoke by his family.

The advanced life support (ALS) team evaluated the patient's carbon monoxide (COHb) level at 30% on peripheral oximetry. Vitals showed significantly elevated blood pressure of 245/137 mmHg, a heart rate of 110 beats per minute, a respiratory rate of 23 breaths per minute, a temperature of 36.6°C, and oxygen saturation of 100% on a non-rebreather mask. Laboratory results revealed a COHb level of 4.1% on venous blood gas (VBG), carbon monoxide level in the blood of 7.1%, brain natriuretic peptide (BNP) level of 489 pg/mL, troponin elevation from 24 pg/mL with a peak of 114 pg/mL, magnesium level of 1.7 mg/dL, and a white blood cell count (WBC) of 12.4 x 10^3^ mm^3^.

**Figure 1:**
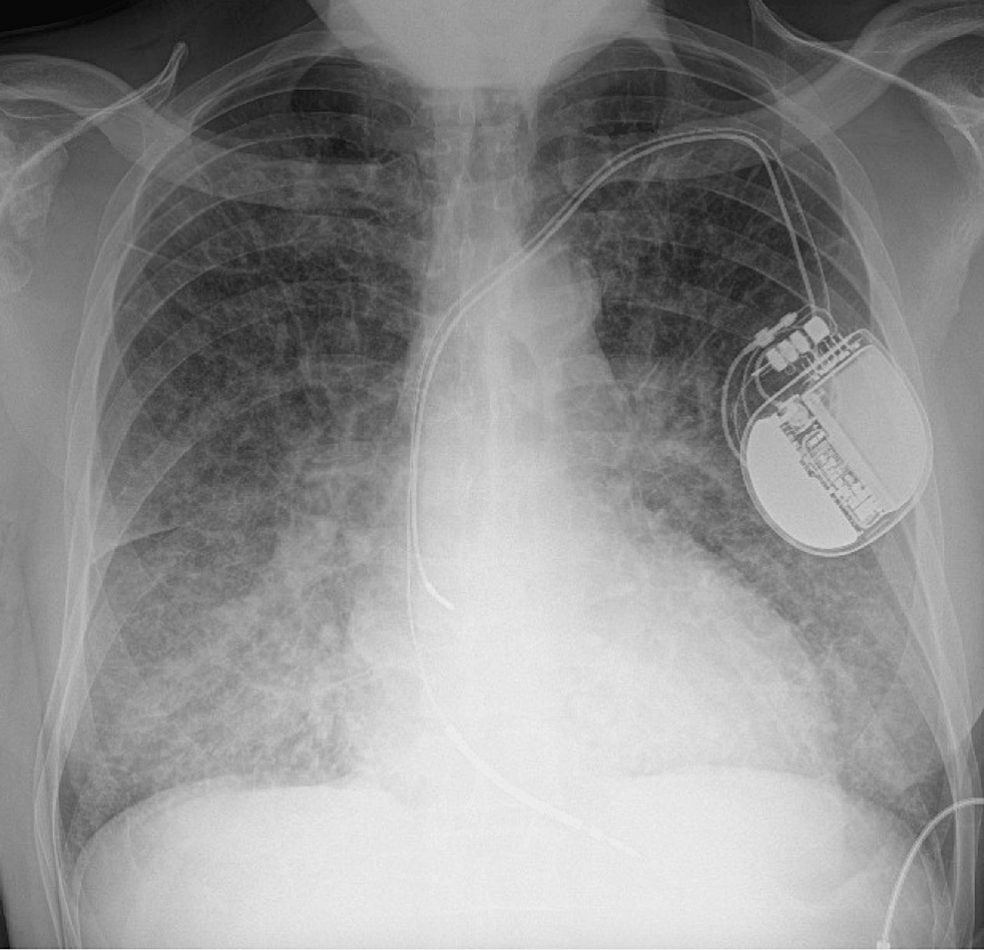
Chest x-ray AP view showing a left-sided ICD and increased broncho-vascular/interstitial lung markings with ill-defined lower lung zones AP - Anteroposterior, ICD - Implantable Cardioverter Defibrillator

**Figure 2:**
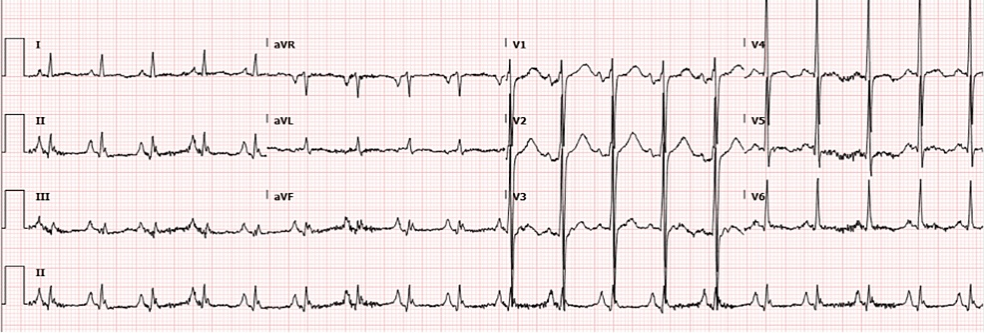
Electrocardiography (EKG) on admission revealing sinus tachycardia, a prolonged QTc interval of 484 ms

The chest x-ray (Figure 1) showed a left-sided ICD and increased broncho-vascular/interstitial lung markings with ill-defined lower lung zones. Electrocardiography (EKG) on admission (Figure 2) revealed sinus tachycardia, a prolonged QTc interval of 484 ms, right atrial enlargement, left ventricular hypertrophy, and q-waves in leads II, III, and avF suggestive of a previous myocardial infarction.

**Figure 3:**
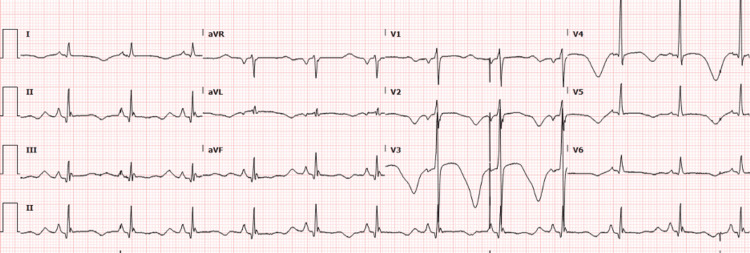
Repeat EKG showing sinus rhythm with a prolonged QTc interval of 612 ms

**Figure 4:**
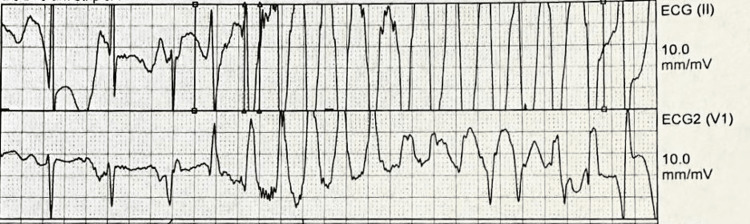
Telemetry rhythm strip showing polymorphic ventricular tachycardia

The patient was admitted to the cardiac care unit (CCU) to manage the hypertensive emergency. Nitroglycerine drip was initiated as part of the treatment plan. The patient began experiencing symptoms of opioid withdrawal, including nausea, profuse sweating, anxiety, and elevated blood pressure, consistent with his endorsement of recent heroin abuse. The Clinical Opiate Withdrawal Scale (COWS) score indicated severe withdrawal (score>10), and buprenorphine 8 mg was administered as per opioid withdrawal protocol. However, subsequent COWS evaluations continued to show scores above eight, requiring two additional doses of buprenorphine 8 mg. Non-opioid alternatives such as trimethobenzamide 200 mg for nausea, clonidine 0.1 mg for hypertension secondary to opioid withdrawal, and hydroxyzine 50 mg for anxiety were also administered due to sustained withdrawal symptoms. A repeat EKG (Figure 3) showed sinus rhythm with a prolonged QTc interval of 612 ms. Figure 4 shows the telemetry strip during the VT episodes.

Overnight, the patient developed sustained PVT with TdP and was given magnesium 2 g IV initially. The ICD device was interrogated, revealing 19 episodes of the R-on-T phenomenon leading to TdP. Two episodes were terminated with anti-tachycardia pacing (ATP), and the patient received 17 shocks. Amiodarone 150 mg IV push and a subsequent amiodarone drip were administered, followed by lidocaine 100 mg IV push and a lidocaine drip upon sustained polymorphic VT. Given sustained polymorphic VT refractory to amiodarone and lidocaine, the decision was made to intubate and sedate the patient as the final resort to control arrhythmia. Propofol and midazolam were used for sedation, and fentanyl pushes for analgesia.

Amiodarone and lidocaine infusions were weaned off upon subsequent resolution of sustained VT. The patient was extubated, and the device's pacing was overridden and reprogrammed to the heart rate of 75 beats per minute. Transthoracic echocardiography (TEE) revealed a left ventricular ejection fraction of 35%-40% and a small pericardial effusion and mitral valve regurgitation. Following the acute episode's resolution and achieving hemodynamic stability, the patient was discharged and advised to follow up with an electrophysiologist for further management.

## Discussion

This case report describes an instance of hydroxyzine-induced TdP in a patient who received hydroxyzine during hospitalization. An extensive literature review of case reports was conducted to provide context and support the findings.

The QT interval, measured from the Q-wave to the end of the T-wave on an electrocardiogram, represents the duration of ventricular repolarization. Corrected QT interval (QTc) accounts for variations in heart rate and is considered abnormal if it exceeds 450 ms in males and 460 ms in females. A QTc longer than 500 ms is associated with a two to three times higher risk of progressing to TdP [7]. When an ectopic beat occurs during prolonged repolarization, known as an R-on-T phenomenon, it can trigger PVT characterized by twisting QRS complexes along an isoelectric line, leading to TdP [7]. Symptoms typically include palpitations, syncope, and dizziness, potentially progressing to ventricular fibrillation and cardiac death in 10% of cases [7].

Hydroxyzine, a first-generation antihistamine, exhibits antiemetic, anxiolytic, and sedative properties. Although it is commonly used to alleviate anxiety symptoms during opiate withdrawal, postmarketing data analysis has associated hydroxyzine with rare cardiac events, including sudden cardiac arrest and TdP, in individuals susceptible to QTc prolongation [2]. This effect is hypothesized to occur due to the drugs’ inhibitory action on the fast-activating potassium (K+) repolarizing channels. Fortunately, this QTc prolongation is rare, and only a handful of cases documented in the literature demonstrate such an association. In 2008, Sakaguchi et al. demonstrated a case of syncope secondary to QTc prolongation in a patient with HERG mutation, which made the patient susceptible to the QTc prolonging effect of hydroxyzine [1]. A similar occurrence occurred in a patient with a complete heart block in whom low-dose hydroxyzine was given for sedation, as documented by Koshino et al. [6]. A review of adverse event reports by Schlit et al. reaffirms the dose-dependent effect of hydroxyzine on QT interval and recommends caution while prescribing hydroxyzine in susceptible individuals [4,6]. One noteworthy characteristic of the study by Schlit et al. is that only patients with susceptibility to QTc prolongation or those taking concurrent QT prolonging drugs were susceptible to torsade at low doses (<100mg of hydroxyzine) [4].

In our literature review, the management approaches for hydroxyzine-induced TdP have varied across cases, ranging from cardiopulmonary resuscitation or drug discontinuation to more aggressive measures. The onset of TdP was observed within 10 minutes to 20 days after initiating therapy, with cumulative doses ranging from 12.5 mg to 225 mg. Further details of the literature review are presented in Table 1.

**Table 1:** Case reports in the literature of hydroxyzine-induced torsades TdP: Torsade de pointes; AF: Atrial fibrillation, HERG: Human Ether-a-go-go–Related Gene, HFpEF: Heart failure with preserved ejection fraction, ICD: Implantable cardioverter-defibrillator

Author, year	Age, Sex	Background	Dosage and reason for administering hydroxyzine	Total cumulative dose of hydroxyzine	Time to onset of TdP after hydroxyzine administration	Additional QTc prolongation agents	QTc interval before TdP	Management of TdP	Outcome
Present case	58, M	Opioid withdrawal, Hypertensive emergency, HFpEF, AICD	50 mg bolus To relieve anxiety during opioid withdrawal	50 mg	3 hours	None	612 ms	MgSo4, amiodarone, lidocaine, intubation, and sedation using propofol, midazolam and fentanyl	Discharged and follow-up with electrophysiologist
Koshini, 2022 [6]	82, F	Dyspnea, bradycardia during exertion ECG showed AV block	12.5 mg bolus For sedation during pacemaker implantation	12.5 mg	10 minutes	None	636 ms	Chest compressions and temporary pacing	Discharge and asymptomatic at follow-up
Acosta-Materan, 2016 [5]	65, F	Palpitations, progressive dyspnea, orthopnea ECG showed AF	25mg/day For a lower extremity pruriginous rash developed during hospital stay	100 mg	4 days of therapy	Flecainide 50mg/12h	-	Cardiac defibrillation 6 times, orotracheal intubation and advanced CPR	Discharged and asymptomatic at follow-up
Sakaguchi, 2008 [1]	34, F	HERG mutation (risk factor for QTc prolongation)	75 mg/day For chronic pruligo	225 mg	3 days of therapy	None	630 ms	Only discontinuation of drug	Asymptomatic on follow-up
Kwon, 1998 [8]	43, M	Fever and rash	12.5 mg/ day For rash	175 mg	14 days of therapy 20^th^ day of hospitalization	None	-	Cardiac defibrillation, CPR, lidocaine, MgSO4 and isoproterenol	Discharged and asymptomatic at follow-up

This case report reinforces the need for caution when using hydroxyzine in patients with a history of or high susceptibility to QTc prolongation. The wide variation of dosage of hydroxyzine also points toward the hypothesis that there may be underlying additional factors that increase the risk of TdP other than the dose dependent response to hydroxyzine. Additionally, our findings highlight the efficacy of deep sedation and endotracheal intubation as termination treatments for refractory VT and PVT that do not respond to standard guideline treatments. This aligns with a study by Bundgaard et al., which also emphasizes the use of deep sedation to terminate refractory VT and PVT [9].

## Conclusions

This case report and literature review emphasizes the importance of careful prescribing practices for QT-prolonging drugs like hydroxyzine to prevent TdP. Healthcare providers should conduct personalized risk-benefit assessments, monitor electrolyte levels, and perform electrocardiograms at baseline and periodically, especially during acute illnesses. Administering the lowest effective dose for the shortest duration, avoiding potential drug interactions, and exercising caution in patients with underlying repolarization abnormalities or a history of TdP is crucial. Furthermore, healthcare providers should exercise caution when prescribing even small doses of hydroxyzine to bradycardic patients, particularly older women with smaller physiques. Adhering to these precautions can help minimize the risk of TdP associated with low-dose hydroxyzine therapy.
